# Placebo and nocebo interventions impact perceived but not actual proprioceptive accuracy

**DOI:** 10.1371/journal.pone.0307072

**Published:** 2024-08-30

**Authors:** Áron Horváth, Blanka Aranyosy, Orsolya Drozdovszky, Attila Szabo, Ferenc Köteles

**Affiliations:** 1 Institute of Psychology, Károli Gáspár University of the Reformed Church in Hungary, Budapest, Hungary; 2 Ádám György Phychophysiology Research Group, Budapest, Hungary; 3 Institute of Health Promotion and Sport Sciences, ELTE Eötvös Loránd University, Budapest, Hungary; 4 Faculty of Health and Sport Sciences, Széchenyi István University, Győr, Hungary; La Sapienza University of Rome, ITALY

## Abstract

Changes in performance caused by positive and negative expectations (i.e., placebo and nocebo responses) were found to play an important role in many aspects of motor performance. This study aimed to test the impact of placebo/nocebo responses and the assumed moderating role of dispositional optimism and anxiety on proprioceptive accuracy, an essential aspect of motor functions. 78 undergraduate university students completed questionnaires assessing dispositional optimism, state anxiety, and motivation to cooperate, then were randomly assigned to three experimental groups. A sham subliminal electric stimulation was applied with claimed positive (placebo group, n = 26), negative (nocebo group, n = 26) or neutral (control group, n = 26) impact on proprioceptive accuracy. Proprioceptive accuracy was measured with active and passive versions of the Joint Position Reproduction task before and after the intervention. Expected and perceived changes in performance were also assessed; changes in state anxiety, optimism, and motivation to cooperate were used as control variables (covariates). Mixed analyses of variance indicated that the experimental manipulation did not affect actual proprioceptive accuracy but impacted expected and perceived performance. Adding the covariates to the models did not substantially change the results. Further, no significant association emerged between actual and perceived change in performance in the active test, and only a weak correlation was found in the passive test. Expected performance did not predict actual performance but predicted perceived performance in both tasks. The results suggest that only perceived (subjective) aspects of proprioceptive accuracy are susceptible to placebo and nocebo interventions.

## 1. Introduction

Generally speaking, placebo and nocebo reactions refer to the consequences of positive (i. e., improvement of a condition) and negative (i. e., worsening of a condition) expectations in various contexts. These phenomena were initially described in the medical field, e.g., for analgesia/hyperalgesia and for the appearance of medication side effects in placebo groups of clinical trials [1–3]; later, it was realized that they play a role in other domains, such as in the effects of psychoactive drugs (alcohol, caffeine), and cognitive performance too [4–7]. Placebo and nocebo responses are triggered by expectations in the broader sense, including conditioning and contextual information [8–11]. Conscious and non-conscious expectations of the brain (called priors) are the critical components of the predictive processing framework [12, 13], the most recent approach to brain mechanisms underlying perception and action [14]. Placebo/nocebo responses refer to both actual (“objective”;e. g., heart rate, blood pressure, or performance in a cognitive or physical task) and perceived (“subjective”;e.g., perceived physical state, mood state, or perceived performance in a task) changes [15]. A number of studies suggest that the latter aspect is typically more pronounced than the former [16–18].

Following the aforementioned theoretical considerations, empirical evidence shows that placebo and nocebo interventions impact various aspects of sports [19, 20] and motor performance [21]. For example, endurance [22, 23], sprint performance [24–26], maximal voluntary strength [27–30] and reaction time [31, 32] can be influenced by the manipulation of expectations. Empirical findings in other physical performance domains, such as balancing ability [21, 33, 34], are less conclusive. Proprioceptive accuracy (PAc), the acuity of perception of the actual state of various components of the motor system (e.g., angle of joints, tension of muscles) [35, 36], is an aspect of motor performance which has not received research attention in the placebo/nocebo field to date [37]. Improving proprioceptive accuracy is an important short-term goal for different warming up [38], stretching [39] and taping techniques [40, 41], and it has a critical role in preventing falls in older adults [42, 43]. Research suggests that the placebo effect plays an essential role in taping techniques that improve different aspects of motor performance [44, 45]. Furthermore, participants’ PAc can be decreased by negative psychophysiological states, such as stress [46]. Overall, findings of the aforementioned studies support the idea that PAc can also be affected by placebo and nocebo manipulations.

Proprioceptive accuracy encompasses several submodalities (i.e. joint position, movement, trajectory, velocity, force, muscle tension, weight and size sense), assessed with different methods [47]. To investigate placebo and nocebo effects on proprioceptive accuracy, we chose the Joint Position Reproduction Test [35], assessing joint position sense, as this is the most widely used and studied method [47], and the measurement is sensitive to the effects of age [36, 48], speed of the trials [49], acute and chronic physical activity, and cognitive functions too [50–52].

Beyond expectations, various psychological factors, e.g., certain trait-like and state-like characteristics, can also influence the placebo and nocebo reaction. A recent systematic review identified dispositional optimism and anxiety as factors that can moderate placebo/nocebo responses [53]. Dispositional optimism, i.e., the generalized tendency to expect positive outcomes, facilitates placebo effects. Anxiety might be dispositional (trait-like) or situation-specific (state). Many trait-anxious people do not participate in nocebo/placebo research [54] because being tested in a laboratory setting induces anxiety, which augments the nocebo response [53], and simultaneously could wipe out a possible placebo response. Indeed, it was recently reported that laboratory-induced expectancy and anxiety, rather than dispositional anxiety, might account to a greater extent for nocebo effects [55]. Furthermore, dispositional anxiety might be facilitative or debilitative, leading to different psychological processes and performance outcomes [56]. Our knowledge of the role of such factors in the field of motor/sports performance is scarce. In an explorative study, Corsi and colleagues [27] found that participants with lower levels of dispositional optimism and higher levels of anxiety showed an increased nocebo response in a task manipulating expectations with respect to the strength of finger pressures (force). Another recent study reported that optimism, anxiety and other psychological characteristics did not impact placebo and nocebo responses in a postural stability task [21]. The controversial findings could be related to the different intervention forms, different placebos, and the fact that some people respond to placebos while others do not [57]. Szabo [58] proposed a model in which learning-based cognition leads to mental evaluation associated with the certainty of the effect of the placebo or nocebo event, agent, object, or information, with more certain (undoubtful) responses leading to stronger effects. However, several psychological factors affect cognition and the subjective appraisal of the causal connection between one’s mental schema and the anticipated outcomes associated with placebos or nocebos. Therefore, these psychological factors should be evaluated to understand why one individual responds in one way while another responds in another to the same research intervention using a placebo/nocebo. In this study, we chose the State and Trait Anxiety Inventory [59, 60] (STAI) and the revised version of the Life Orientation Test [61, 62] (LOT-R) to assess anxiety and optimism respectively, because these are widely used, validated and reliable questionnaires, that are also adapted to the Hungarian language.

The primary objective of the present study was to explore the impact of placebo and nocebo interventions on PAc. We hypothesized that a placebo manipulation increases, whereas nocebo manipulation decreases actual (*H1a*) and perceived (*H1b*) performance in a task assessing PAc concerning the position of the elbow joint. Also, we expected that dispositional optimism would show a positive association with the placebo and a negative association with the nocebo response (*H2*), and state anxiety would negatively impact the placebo response and enhance the nocebo response (*H3*).

## 2. Materials and methods

The study has been preregistered at https://osf.io/rebqm. The data and the analysis are available at: https://osf.io/khuvb/.

### 2.1. Participants

With convenience sampling method, 78 undergraduate students (83% female, mean age: 21.25±3.86) of the Eötvös Loránd University were included in the study. The participation was voluntary, compensated with partial course credit (multiple participation opportunities were offered to the students and the course could be completed without participation in any empirical study). Sample size calculation was carried out using the G*power software [63]. Based on the findings of Hurst and colleagues [20], the predicted effect size was f = 0.180. 78 participants were required to reach an alpha level of 0.05 and a power of 0.8 to test a two-way interaction in a mixed analysis of variance (two-factorial ANOVA; one between-subject factor with three levels and one within-subject factor with two levels). The experimental procedure was approved by the Research Ethics Committee of the Eötvös Loránd University (approval number: 2022/428). Participants signed a written informed consent before the experiment, confirming that they were not treated for a neurological or psychiatric condition, and were not under the influence of alcohol or any psychoactive substances. Data was collected and recorded anonymously. They were randomized with the help of the sample() function of the R software [64] into the three groups (placebo, nocebo and control) in a 1:1:1 ratio. Recruitment period for this study lasted from the 7^th^ of November 2022 to august 31^st^ of August 2023.

### 2.2. Proprioceptive accuracy

Proprioceptive accuracy in the dominant elbow joint was assessed with the Joint Position Reproduction Test (JPR) [35]. We used a custom-made motorized device [65], to move and measure joint positions with a precision of 0.1 degrees. We applied two conditions: active and passive reproduction, always in a random order. In both conditions, the joint was moved passively (by the machine) to a target position, held there for some time, then moved back to starting position. From there, participants had to replicate the target position in the active condition by actively moving their arms. In the passive condition, the motorized device started to move the arm toward the target position, and participants had to indicate when it reached it. In both conditions, a button press was used to signal the replicated position. The equipment was adjusted to the length of the participant’s arms. We used a combination of different starting positions, target positions, movement speeds, and times spent in the target position. The starting position was 30 or 150 degrees, the target position was 60, 90, or 120 degrees, the movement speed was 12 or 24 degrees/second, and the time spent in the target position was 2 or 4 seconds. We presented each combination once (2 starting positions * 3 target positions * 2 movement speeds * 2 times spent in target position) in random order, resulting in 24 trials per condition. To determine performance (absolute error score), the mean of the absolute values of the differences between presented and replicated positions was calculated. Higher values of absolute error refer to lower levels of proprioceptive accuracy. To determine the change in actual performance, we subtracted the pre-intervention absolute error from the post-intervention absolute error, higher scores referring to an increment in absolute error. Indices were calculated separately for the active and for the passive test. Cronbach’s alpha values were acceptable in every condition (active pre-measurement: 0.78, active post-measurement: 0.82, passive pre-measurement: 0.70, passive post-measurement: 0.66). Systematic error score, another widely used measure of PAc, was not used due to the very low internal consistency of the indices.

### 2.3. Questionnaires and questions

#### 2.3.1. Expected performance change

Expected change in performance was assessed with a 100-mm Visual Analogue Scale (VAS). Participants had to rate the following question: “*In what direction and to what extent do you think your performance will change compared to the baseline*?”. The two endpoints were: “*significant deterioration*” and “*significant improvement*” with the middle point “*will not change*”. The theoretical range of the scale was 0–100, where higher scores indicated higher expected improvements.

#### 2.3.2. Perceived performance change

Perceived performance was assessed with a 100-mm VAS. The question was: *“In what direction and to what extent do you think your performance has changed compared to the baseline*?*”*. The two end points were: “*significantly deteriorated*” and “*significantly improved*”, and the middle point was “*did not change*”. The theoretical range of the scale was 0–100, where higher scores indicated higher perceived improvements.

#### 2.3.3. Motivation to cooperate

Participants’ motivation to cooperate (CoMotiv) was assessed with six items [66] (Szemerszky et al., 2010). Statements (e.g., “*I am happy to participate in this study*”, “*I found the experimenter sympathetic*”) were answered on 5-point Likert scales. Higher values on the scale refer to a higher motivation to cooperate. Internal consistensy of the instrument was acceptable in the original Hungarian version (Cronbach’s alpha = 0.63) [66] and in this sample (Cronbach’s alpha = 0.76).

#### 2.3.4. Optimism

The Hungarian version of the Life Orientation Test Revised (LoT-R) [62] was used to assess dispositional optimism. Ten items (e.g., “*I’m always optimistic about my future*”, “*In uncertain times*, *I usually expect the best*”) were rated on a five-point Likert scale. The scale includes 4 filler items; therefore the total score was calculated from the six assessment items. Higher values on the scale refer to higher levels of dispositional optimism. The Hungarian version proved to be valid and psychometrically sound (Cronbach’s alpha = 0.784), and internal consistency was also acceptable in this sample (Cronbach’s alpha = 0.78).

#### 2.3.5. State anxiety

The Hungarian state version of the State and Trait Anxiety Inventory (STAI-S) [67] was used to assess the current anxiety level of the participants before and after the intervention. The Hungarian version proved to be valid and psychometrically sound (Cronbach’s alpha was between 0.81 and 0.93) [67]. 20 questions (e.g., “*I am tense*”, “*I feel nervous*”) were rated on a 4-point Likert-scale. Higher total scores refer to higher levels of state anxiety. Participants filled out the questionnaire before and after the experimental manipulation. Internal reliability of the scale was good (Cronbach’s alpha = 0.91 for both pre- and post-intervention scores). We subtracted the pre-intervention scores from the post-intervention scores to calculate the change in state anxiety (STA-S change). Higher values referred to increased anxiety levels after the intervention.

### 2.4. Procedure

The study was a randomized, single-blinded experiment applying a mixed design, i.e., a within-subject dimension (pre- and post-intervention measurements) and a between subject dimension (placebo, nocebo and control groups). After arriving at the laboratory, participants signed the informed consent form, filled out the questionnaires assessing optimism, motivation to cooperate, and pre-intervention state anxiety, and completed the pre-intervention proprioceptive accuracy measurement. Subsequently, they were randomly assigned to one of the three experimental conditions. The groups were labeled with letters; thus, the experimenter wasn’t aware of which condition the participants were assigned to. Two electrodes were placed on the ventral side of the participants’ left arm, 7 cm below and above the crook of the arm. The machine administering sham electric stimulation was connected to the electrodes and visibly turned off in this phase. In the next step, participants received information about the respective intervention from an audio file (to keep the experimenter blind, they listened to the instructions through headphones). The placebo group was told that they would receive subliminal electric stimulation, which improves proprioceptive accuracy; the nocebo group received the information that the stimulation would decrease proprioceptive accuracy, and the control group was told that they would not receive any effective stimulation, so their proprioceptive accuracy would not be affected. In conclusion, the study was blind from the viewpoint of the experimenter but could not be blind from the viewpoint of the participants. After listening to the instructions, they filled out the post-intervention state anxiety questionnaire. The experimenter turned on the sham stimulation device and started the claimed intervention protocol. The device was turned on during the second proprioceptive accuracy assessment, but no stimulation occurred. In a random order, participants completed the passive and active proprioceptive accuracy test; expected and perceived performance were assessed before and after the tests. Finally, the experimenter turned off the device; then removed the electrodes, and participants were asked about their experience, including the suspected goal of the experiment. (They were asked the questions “*Did you feel anything from the stimulation*?” and “*How do you think it affected your performance*?”). Based on their answers to the questions, none of them thought that the stimulation was not real. An experimental session lasted for approximately an hour.

### 2.5. Statistical analysis

Statistical analysis was conducted with SPSS v.26 software (IBM Corporation, New York). For the proprioceptive accuracy tests extreme outliers (outside three interquartile ranges) were removed per trial, based on box plots. Differences within groups with respect to pre-intervention absolute error scores and expected and perceived performance were estimated with one-way analyses of variance (ANOVA). Here we expected no difference in absolute error scores (homogeneity check), but significant differences between expected (manipulation check) and perceived performance (H1b), the placebo group showing the highest and the nocebo group showing the lowest values. Change in actual performance (i.e. proprioceptive absolute error) was tested separately for the active and passive version of JPR, with 3*2 mixed ANOVAs (between-subject factor: group (placebo, nocebo or control); within-subject factor: time (pre- and post-intervention measurement). We predicted a significant time*group interaction, with an increment of absolute error in the nocebo group, a decrement in the placebo group, and no change in the control group between pre- and post-intervention measurement (H1a). As we hypothesized that actual and perceived performance changes can be explained by expectations, a significant, negative association was hypothesized between expected performance change and actual performance change (higher levels of proprioceptive error refer to worse PAc) and a significant positive association was predicted between expected and perceived performance change (dose-response relationship). The contribution of optimism and anxiety change was checked with mixed model analyses of covariance (ANCOVA); in these models, one questionnaire score was entered in the respective mixed ANOVA as a covariate. A three-way interaction was expected between time, group and the covariates. We hypothesized that optimism shows a negative correlation with the increment of absolute error in the placebo and nocebo, but not in the control group after the intervention (H2). In a similar vein, an increment in state anxiety was predicted to have a positive correlation with the increment of absolute error scores post intervention in the placebo and nocebo groups (H3). To control for a possible bias due to compliance, we also checked if there is a three-way interaction between group, time and motivation to cooperate. For more clarity, we only present results in the manuscript that are relevant to the testing of hypothesis or are significant. Every statistical result can be accessed openly at https://osf.io/khuvb/. Tukey-corrected *p*-values were used in the *post hoc* analysis. Associations between proprioception-related variables were estimated with Pearson correlation.

## 3. Results

### 3.1. Active condition

#### 3.1.1. Homogeneity of the groups and experimental manipulation

**Fig 1:**
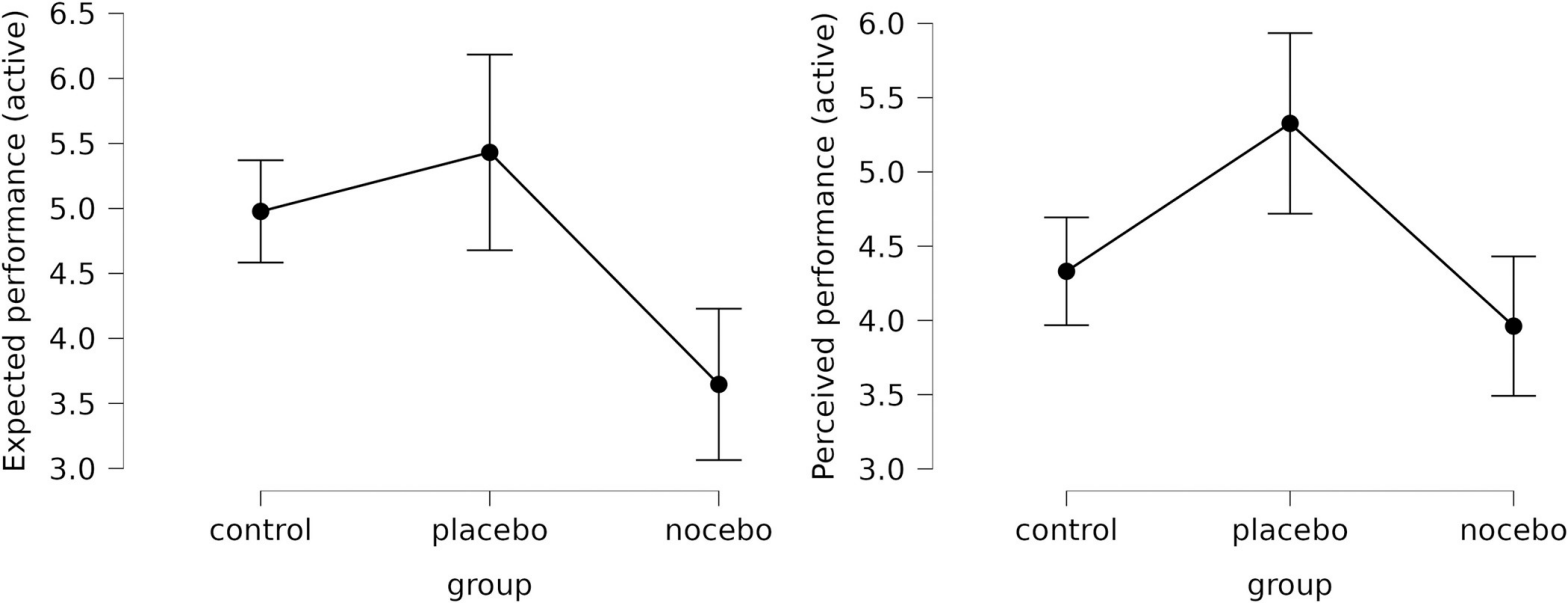
Participants’ expected and perceived performance in the three experimental groups (active condition). Error bars represent 95% confidence intervals.

Descriptive statistics are presented in Table 1. There were no differences between the three groups in baseline absolute error ((*F*(2,75) = 0.867; *p* = .424; *η*^2^ = .023), indicating homogeneity of the groups. ANOVA indicated significant differences between the expected performance of the three groups with a large effect size (*F*(2,75) = 10.326; *p* < .001; *η*^2^ = .216); according to the post hoc analysis (*p*_*Tukey*_ < .05), expectations of the nocebo group were significantly lower than those of the control and placebo group (Fig 1), indicating that the experimental manipulation was partially successful.

**Table 1:** Descriptive statistics (*M±SD*) of the assessed variables in the three groups in the active condition.

	Control	Placebo	Nocebo
Expected performance	4.98±0.97	5.43±1.86	3.65±1.44
Baseline absolute error (°)	5.84±1.53	5.73±1.45	6.33±2.16
Post-intervention absolute error (°)	5.91±1.67	5.76±1.78	6.35±2.37
Perceived performance	4.33±0.90	5.33±1.51	3.96±1.16

#### 3.1.2. Actual and perceived performance (H1a, H1b)

The mixed model ANOVA indicated no significant time x group (*F*(2,75) = .018; *p* = .982; *η*_*p*_^*2*^ < .001) interaction for absolute error (i.e., H1a was not supported). However, perceived performance was better (*p*_*Tukey*_ < .05) in the placebo group than in the other two groups (*F*(2,75) = 8.789; *p* < .001; *η*_*p*_^*2*^ = .190; Fig 1), meaning that H1b was partially supported, as there was no significant (p>.05) difference between the nocebo and control group.

#### 3.1.3. Associations between performance-related variables

Participants’ expectations did not predict their actual performance change (*r* = .01, *p* = .937); however, there was a medium level association (*r* = .31, *p* = .005) between expected and perceived performance change. Actual performance change was not significantly related to perceived performance change (*r* = .01, *p* = .384).

#### 3.1.4. Questionnaires

Descriptive statistics of the questionnaires are presented in Table 2. Concerning dispositional optimism, no significant time x group x LoT-R interaction (*F*(2,71) = 0.868; *p* = .424; *η*_*p*_^*2*^ = 0.024) were found, indicating that optimism did not modify the experimental manipulation (H2 was not supported). The main effect of LoT-R was significant (*F*(1,71) = 5.111; *p* = .027; *η*_*p*_^*2*^ = .067). To investigate this effect, we took the mean of pre- and post-intervention absolute error scores, and tested the correlation with LOT-R. Correlation analysis indicated a weak, positive association (r = 0.23, p = 0.41); in other words, higher level of dispositional optimism was associated with worse performance.

**Table 2:** Descriptive statistics of the assessed questionnaire variables.

	N	M	SD	min	max
LoT-R	77	22.0	4.27	13	28
STAI-S change	76	-2.0	4.12	-13	7
CoMotiv	78	27.5	2.53	21	30

Note: Lot-R: Life Orientation Test-Revised; STAI-S: State and Trait Anxiety Inventory State scale; CoMotiv: Motivation to cooperate

As for change in anxiety, no significant time x group x STAI-S change interaction (*F*(2,70) = 0.674; *p* = .513; *η*^2^ = .019) were found, meaning that the change in anxiety does not modify the effect of the experimental manipulation (H3 was not met).

When including participants’ CoMotiv score, we found a significant time*group*CoMotiv (*F*(2,72) = 5.185, *p* = .008, *η*_*p*_^*2*^ = .126) interaction. To explain the three-way interaction, a correlation analysis was conducted between actual performance change and CoMotiv score separately for the three experimental groups. In the placebo group, a positive, medium level correlation was found (*r* = .54, *p* = .005), while no significant correlation (*p*>.05) was revealed in the nocebo and the control group. The association in the placebo group means that higher levels of CoMotiv were associated with a greater reduction in performance, meaning that contrary to what could have been expected, those with higher levels of motivation to cooperate showed a greater reduction in performance in the placebo group. The time*group interaction was also significant (*F*(2,72) = 5.134, *p* = .008, *η*_*p*_^*2*^ = .125) after the inclusion of CoMotiv, but post-hoc tests did not reveal significant differences between groups at pre or post-measurement.

### 3.2. Passive condition

#### 3.2.1. Homogeneity of the groups and experimental manipulation

**Fig 2:**
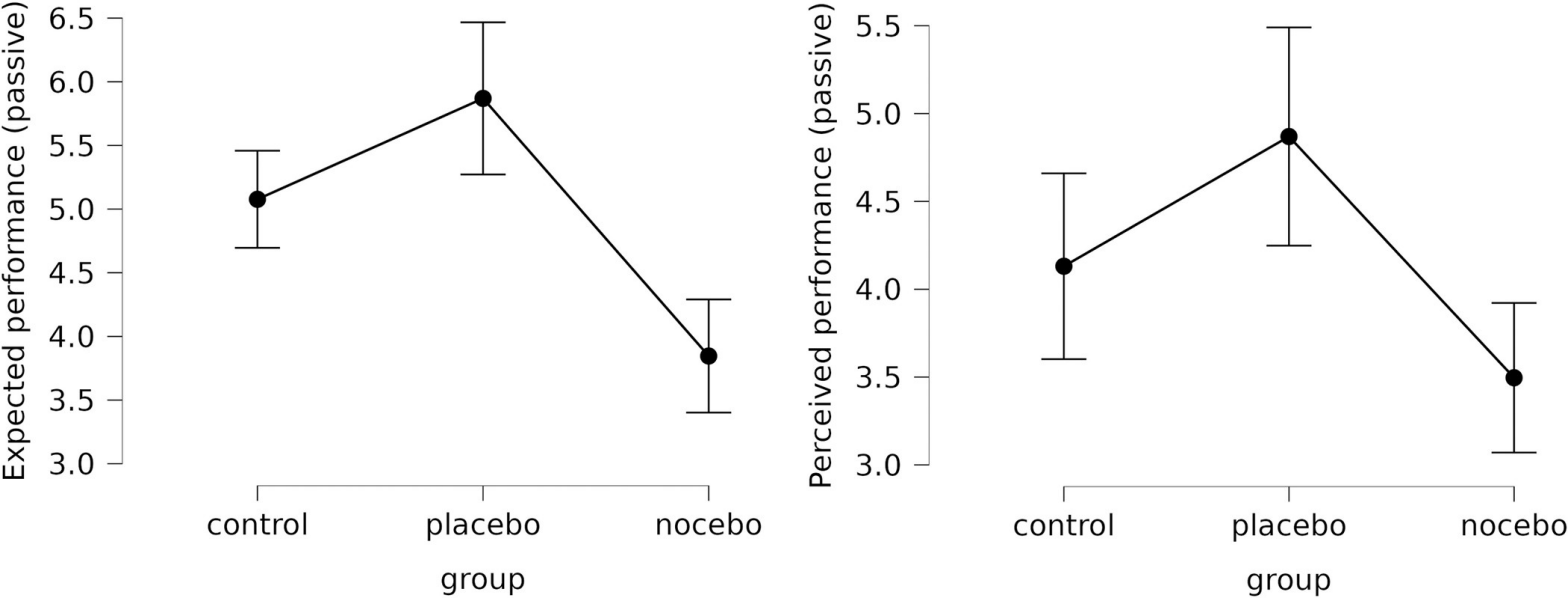
Participants’ expected and perceived performance change in the three experimental groups (passive condition). Error bars represent 95% confidence intervals.

Descriptive statistics are presented in Table 3. There were no differences between the three groups in baseline proprioceptive accuracy (*F*(2,75) = 0.795; *p* = .455; *η*^2^ = .021), indicating that the groups are homogeneous. The ANOVA indicated significant differences between the expectations of the three groups with a large effect size (*F*(2,75) = 18.896; *p* < .001; *η*^2^ = .335); *post hoc* analysis (*p*_*Tukey*_ < .05) indicated significant differences between the three groups in the expected directions (Fig 2), meaning that the experimental manipulation was fully successful.

**Table 3:** Descriptive statistics (*M±SD*) of the assessed variables in the three groups in the passive condition.

	Control	Placebo	Nocebo
Expected performance	5.08±0.94	5.87±1.48	3.85±1.10
Baseline absolute error (°)	5.81±1.63	6.03±1.45	6.43±2.25
Post-intervention absolute error (°)	-0.40±2.07	0.54±1.91	-0.65±1.61
Perceived performance	4.13±1.31	4.87±1.54	3.50±1.05

#### 3.2.2. Actual and perceived performance change (H1a, H1b)

The mixed model ANOVA indicated no significant time x group (*F*(2,75) = 2.920; *p* = .060; *η*_*p*_^*2*^ = .072) interaction for absolute error in the passive assessment, indicating no effect of the experimental manipulation (H1a was not met). Concerning perceived performance change, ANOVA indicated significant differences (*F*(2,75) = 7.097; *p* = .002; *η*^2^ = .159); the nocebo group showed significantly (*p*_*Tukey*_ < .05) worse performance than the placebo group (Fig 2), meaning that H1b was partially supported, as placebo and nocebo groups did not differ from the control group (p>.05).

#### 3.2.3. Associations between performance-related variables

Participants’ expectations were unrelated to their actual performance change (*r* = .12, *p* = .306); however, there was a medium-level association (*r* = .46, *p* < .001) between expected and perceived performance. Actual performance change was weakly related to perceived performance (*r* = .23, *p* = .040).

#### 3.2.4. Questionnaires

For dispositional optimism, no time x group x LoT-R interaction (*F*(2,71) = 0.442; *p* = .645; *η*_*p*_^*2*^ = .012) were found, indicating no effect of optimism on the experimental manipulation (H2 was not met). A significant group x LoT-R interaction was revealed (F(2,71) = 3.285, p = .043, *η*_*p*_^*2*^ = .085). To explain this, we took the mean of pre and post-intervention absolute error scores, and then tested the correlation between this score and LOT-R separately in the three groups. No statistically significant correlations emerged between the variables in the nocebo and control group (*p*>.05), but a significant correlation was found in the placebo group (r = 0.42, p = 0.32), indicating that a higher level of optimism is associated with worse performance in the placebo group.

For change in anxiety, no significant time x group x STAI-S change interaction (*F*(2,70) = 1.505; *p* = .229; *η*_*p*_^*2*^ = .041) were found, indicating no effect of change in anxiety for the experimental manipulation (H3 was not met). Time x group interaction became significant after the inclusion of STAI-S change score (*F*(2,70) = 3.234; *p* = .045; *η*_*p*_^*2*^ = .085). However, post hoc analysis did not reveal significant differences between the groups at pre- and post-measurement.

When considering motivation to cooperate, we found no significant time*group*CoMotiv (*F*(2,72) = 1.993, *p* = .144, *η*_*p*_^*2*^ = .052) interaction, indicating that motivation to cooperate did not modify the effect of the experimental manipulation.

## 4. Discussion

In this laboratory study, which tested 78 young individuals, placebo and nocebo manipulations using sham subliminal electric stimulation of the elbow joint with claimed positive and negative effects on accuracy, respectively, did not change the participants’ actual performance either in active or passive movement-led proprioceptive accuracy tests. However, significant changes were found in perceived performance, which can be explained partly by the participants’ expectations. Dispositional optimism, state anxiety, and participants’ motivation to cooperate did not substantially impact the results. These findings suggest that proprioceptive accuracy is unlikely to be affected by information-based (deceptive) electrical stimulation, however expectations affect the perceived performance. Therefore, in clinical and research settings, perceived performance is susceptible to bias, and consequently, when making important decisions, objective assessment is necessary.

Participants’ expectations in the three groups indicated that the nocebo manipulation worked well for both active and passive conditions, whereas the placebo manipulation was only effective in the passive condition. However, the difference between the effects of placebo and nocebo manipulations was significant in both conditions though. Considering the large effect sizes, these patterns suggest that the experimental manipulation was mostly successful shifting participants’ expectations in the assumed direction. Passive placebos are rarely used in research; therefore, comparing our results to other studies is difficult, especially in the context of the placebo used and the associated intervention. However, our results are comparable to a study using active and passive (placebo) cycling [68] that found no improvement in either condition’s dependent measures (mood and working memory). In this study, passive cycling was employed as a placebo control to active cycling, whereas in our study, the passive condition was implemented as a means of control for an active intervention, both being associated with different information.

Participants’ overall baseline performance, manifested in a mean proprioceptive accuracy value of about 6°, matched the accuracy reported in other studies using the same paradigm [65, 69, 70]. Our results suggest that placebo/nocebo manipulations cannot significantly influence this accuracy. The finding that expectation was not significantly related to actual performance change in both conditions lends further support to this idea. A possible explanation for these results is that proprioceptive accuracy, as opposed to many other aspects of physical performance, primarily relies on a sensory process, not a motor process. Also, lack of attentional resources can negatively impact sensory accuracy; for example, a cognitively demanding secondary task deteriorated PAc [50–52]. However, if the environment is not distracting and participants can focus on the task, no decrease in performance will occur.

In contrast to the actual performance, participants’ perceived performance showed differences between groups. The placebo group was characterized by better perceived performance than the other two groups in the active condition, and the nocebo group’s perceived performance was worse than that of the placebo group in the passive condition. These findings match the results of an identical research design using an inert nasal spray [32] for placebo/nocebo agents and cognitive tasks as the dependent measures. In this work, positive or negative performance expectations did not lead to changes in *actual* performance, but those who believed they received a stimulating spray reported significantly better-perceived performance than those who believed they were given a performance-deteriorating spray. A similar pattern was found in postural stability [21], and the current research finding also suggests that while manipulating performance expectations might influence perceived changes in performance, it does not affect the actual perceptual or cognitive performance in healthy adults. These findings could explain why many people consume nutritional supplements that have no or potentially detrimental effects on health [71].

Based on the above discussion, our study showed that expectations positively correlated with perceived performance in active and passive conditions. These findings suggest that expectations also play a substantial role in the perception of proprioceptive accuracy. Such an impact can be assumed in tasks where actual performance cannot be accurately detected by participants [21], and the low correlation values between actual and perceived performance indicate that this was the case in the current study. Other studies also showed that the association between actual and perceived performance is typically very weak in proprioceptive and interoceptive accuracy tasks [21, 65, 72]. As stated in the predictive processing framework, the lack of accurate (bottom-up) information about performance enhances the contribution of top-down factors, such as expectation, to the perception of performance [73, 74]. In other words, participants in such cases are prone to experience what they expected.

This study assessed three self-reported characteristics: participants’ motivation to cooperate, dispositional optimism, and state anxiety. The first construct was included to control for the impact of demand characteristics. Thus, higher motivation to cooperate was expected to be associated with stronger placebo and nocebo responses in the respective groups. Our analysis did not support this assumption, suggesting that our findings cannot be considered experimental artifacts. Controlling state anxiety was important because stress can decrease proprioceptive accuracy [46], and receiving a claimed performance worsening electric stimulation may cause an increased anxiety level for the nocebo group. Also, anxiety is usually related to weaker placebo and more potent nocebo effects [27, 53, 75]. Optimism was included, as it was found to be related to stronger placebo and weaker placebo responses [27, 53, 75]. In statistical terms, a three-way interaction between group, time, and the covariant would show such an effect. From the three variables, a three-way interaction was found only for the motivation to cooperate in the active condition of the proprioceptive task (see above). Similarly, unexpected results emerged for optimism, showing that higher levels of optimism were associated with generally worse performance in the active condition. In the passive condition, optimism was also related to a worse performance, but only in the placebo group. These findings are difficult to explain; however, it can be concluded that neither anxiety nor optimism played the expected role in developing proprioceptive accuracy-related placebo/nocebo responses.

The scope of the study was to manipulate expectations associated with proprioception. In this work such intervention yielded perceived (“subjective”) but not actual (“objective”) effects. Future studies could improve the investigation of this subject by using a within-participants research design, other placebo agents with higher perceived effectiveness such as sham injection or inert pill and determining *a priori* whether the participant is a placebo responder or not. The implication of these findings relates to how information from various sources might influence positively or negatively proprioception in real life situations.

### 4.1. Limitations

Perhaps the most critical limitation of the study concerns the placebo/nocebo manipulation we administered. Although differences in participants’ expectations showed that the manipulation was largely successful, more powerful interventions leading to more polarized expectations could have caused changes in actual performance. Also, considering the particular sample (young individuals), these findings should be cautiously generalized. Finally, despite no differences at baseline, another possible limitation is that different participants in three groups may respond differently to the various interventions and associated instruction, which might be why 20 of 32 studies in the review of Hurst and colleagues [20] used a within-participants research design.

### 4.2. Conclusions

In conclusion, proprioceptive accuracy concerning the position of the elbow joint is not malleable to placebo/nocebo manipulations. However, the perception of performance is affected by such interventions. Also, real and perceived performance showed no or only weak association. An essential message for practitioners is that top-down factors are more likely to influence subjective reports of changes in proprioceptive accuracy. Furthermore, in medical settings, the objective assessment of proprioceptive accuracy is necessary since the subjective reports might be affected by placebo and nocebo effects. Based on the current findings, such tests appear unaffected by anxiety, optimism, and the cooperative effort of the people taking part in proprioceptive accuracy measurements.
